# Supplementary material for: The Moderating Role of Personality on the Effects of Concentration-, Ethics- and Wisdom-Based Meditation Practices for Well-Being and Prosociality
Source: Healthcare (Basel). 2025 Nov 25;13(23):3044. doi: 10.3390/healthcare13233044 (PMC12692481; doi:10.3390/healthcare13233044)
Supplement: Supplementary file 1 [file healthcare-13-03044-s001.zip › healthcare-3935685-supplementary.pdf]

Supplementary File S1. Descriptive Statistics for ANCOVA

Means and Standard Deviations of prosocial outcomes (PSA) pre- and post-intervention by personality trait category (high vs. low).

Personality	High/Low	Group		Pre-		Post-	
			n	**Mean**	**SD**	**Mean**	**SD**
Neuroticism	High	MBI-CE	12	40.83	7.57	36.17	9.33
		MBI-CEW	10	28.4	8.34	37.4	7.57
		Control	8	38	14.12	35.88	12.18
	Low	MBI-CE	9	34.22	9.87	34.33	11.79
		MBI-CEW	8	40	9.24	37.88	4.94
		Control	9	37.44	12.76	37	9.91
Agreeableness	High	MBI-CE	7	34.14	11.6	31.57	7.14
		MBI-CEW	8	31.12	12.04	35.75	5.09
		Control	9	32.56	11.84	31.67	10.58
	Low	MBI-CE	14	39.93	7.18	37.29	11.19
		MBI-CEW	10	35.5	8.96	39.1	7.13
		Control	8	43.5	12.36	41.88	8.39
Conscientiousness	High	MBI-CE	8	35.25	8.45	37.5	11.65
		MBI-CEW	10	33.2	11.36	37.6	6.83
		Control	9	37.56	14.68	36.33	11.94
	Low	MBI-CE	13	39.69	9.3	34.08	9.48
		MBI-CEW	8	34	9.7	37.62	6.19
		Control	8	37.88	11.8	36.62	9.93
Openness	High	MBI-CE	16	39.44	9.22	36.5	11.1
		MBI-CEW	7	25.29	3.99	34	3.51
		Control	17	37.71	12.99	36.47	10.7
	Low	MBI-CE	5	33.4	7.44	31.8	6.22
		MBI-CEW	11	38.82	9.71	39.91	6.82
		Control	0	N/A	N/A	N/A	N/A
Extraversion	High	MBI-CE	7	37.71	11.54	35.57	11.53

		MBI-CEW	7	37	11.65	38.43	5.44
		Control	8	38	14.12	35.88	12.18
	Low	MBI-CE	14	38.14	8.01	35.29	9.96
		MBI-CEW	11	31.36	9.34	37.09	7.09
		Control	9	37.44	12.76	37	9.91

Means and Standard Deviations of psychological well-being outcomes (PWB) pre- and post-intervention by personality trait category (high vs. low).

**Personality**	**High/Low**	**Group**		**Pre-**		**Post-**	
			n	**Mean**	**SD**	**Mean**	**SD**
Neuroticism	High	MBI-CE	12	68.83	5.2	71.17	2.41
		MBI-CEW	11	67.91	7.76	71.27	5.39
		Control	8	38	14.12	35.88	12.18
	Low	MBI-CE	9	68.67	7.5	71.44	7.4
		MBI-CEW	7	69.86	5.55	72.57	4.69
		Control	9	34.44	12.61	42.78	11.65
Agreeableness	High	MBI-CE	11	68.82	7.22	72.36	5.9
		MBI-CEW	7	68.14	6.52	72.57	6.21
		Control	8	31.62	10.14	33.38	11.31
	Low	MBI-CE	10	68.7	5.01	70.1	3.75
		MBI-CEW	11	69	7.39	71.27	4.36
		Control	9	40.11	14.56	45	10.36
Conscientiousness	High	MBI-CE	8	69	7.23	69.88	3
		MBI-CEW	10	69.6	8.02	70.1	3.51
		Control	9	34.56	14.58	42.11	13.73
	Low	MBI-CE	13	68.62	5.64	72.15	5.87
		MBI-CEW	8	67.5	5.42	73.88	6.03
		Control	8	37.88	11.8	36.62	9.93
Openness	High	MBI-CE	16	67.5	5.85	69.44	2.48
		MBI-CEW	7	68.14	6.52	72.57	6.21

		Control	17	36.12	13.04	39.53	12.06
	Low	MBI-CE	5	72.8	5.63	77.2	6.72
		MBI-CEW	11	69	7.39	71.27	4.36
		Control	0	N/A	N/A	N/A	N/A
Extraversion	High	MBI-CE	7	67.14	6.62	70.86	3.72
		MBI-CEW	7	67.86	5.55	71.86	3.72
		Control	10	37.7	15.2	38.5	13.45
	Low	MBI-CE	14	69.57	5.93	71.5	5.67
		MBI-CEW	11	69.18	7.82	71.73	5.88
		Control	7	33.86	9.84	41	10.58

Supplementary File S2. Moderation effects using median split for ANCOVA and Planned Contrasts

Moderation of prosocialness outcomes by personality traits.

Personality	ANCOVA interaction (F, p, η²)	Cell sizes (High)	Cell sizes (Low)	Planned contrasts (High)	Planned contrasts (Low)
Neuroticism	F(2,49)=3.37, p=0.043, η²=0.121	C n=8, CE n=12, CEW n=10	C n=9, CE n=9, CEW n=8	CEW–CE Δ=13.67 [7.02,20.31], p=0.00052, g=1.69; CEW–C Δ=11.13 [7.53,14.72], p=6.6e-06, g=2.86.	CEW–CE Δ=−2.24 [−9.46,4.99], p=0.519, g=−0.30; CEW–C Δ=−1.68 [−8.43,5.07], p=0.603, g=−0.24
Agreeableness	F(2,49)=0.23, p=0.799, η²=0.009	C n=9, CE n=7, CEW n=8	C n=8, CE n=14, CEW n=10	CEW–CE Δ=7.20 [−3.90,18.29], p=0.184, g=0.68; CEW–C Δ=5.51 [−3.14,14.17], p=0.183, g=0.70.	CEW–CE Δ=6.24 [0.06,12.42], p=0.0479, g=0.77; CEW–C Δ=5.23 [−0.98,11.43], p=0.092, g=0.84
Conscientiousness	F(2,49)=1.43, p=0.250, η²=0.055	C n=9, CE n=8, CEW n=10;	C n=8, CE n=13, CEW n=8	CEW–CE Δ=2.15 [−6.79,11.09], p=0.617, g=0.23; CEW–C Δ=5.62 [−2.34,13.58], p=0.154, g=0.64.	CEW–CE Δ=9.24 [3.18,15.31], p=0.0048, g=1.21; CEW–C Δ=4.88 [0.38,9.37], p=0.0357, g=1.11
Openness	F(2,50)=0.39, p=0.677, η²=0.015	C n=17, CE n=16, CEW n=7;	C n=0, CE n=5, CEW n=11	CEW–CE Δ=11.65 [5.04,18.26], p=0.00157, g=1.32; CEW–C Δ=9.95 [4.53,15.37], p=0.00204, g=1.81.	CEW–CE Δ=2.69 [−8.13,13.51], p=0.572, g=0.32; CEW–C not estimable
Extraversion	F(2,49)=0.21, p=0.811, η²=0.009	C n=8, CE n=7, CEW n=7;	C n=9, CE n=14, CEW n=11	CEW–CE Δ=3.57 [−6.71,13.85], p=0.464, g=0.38; CEW–C Δ=3.55 [−4.82,11.92], p=0.351, g=0.51.	CEW–CE Δ=8.58 [1.91,15.26], p=0.0140, g=0.99; CEW–C Δ=6.17 [−0.10,12.45], p=0.0534, g=0.90

Moderation of well-being outcomes by personality traits

Personality	ANCOVA interaction (F, p, η²)	Cell sizes (High)	Cell sizes (Low)	Planned contrasts (High)	Planned contrasts (Low)
Neuroticism	F(2,49)=1.94, p=0.155, η²=0.073	C n=8, CE n=12, CEW n=11	C n=9, CE n=9, CEW n=7	CEW–CE Δ=1.03 [−6.00,8.06], p=0.759, g=0.13; CEW–C Δ=5.49 [−1.33,12.30], p=0.105, g=0.68.	CEW–CE Δ=−0.06 [−6.57,6.44], p=0.984, g=−0.01; CEW–C Δ=−5.62 [−18.02,6.78], p=0.339, g=−0.43
Agreeableness	F(2,49)=3.60, p=0.035, η²=0.128	C n=8, CE n=11, CEW n=7	C n=9, CE n=10, CEW n=11	CEW–CE Δ=0.88 [−6.96,8.73], p=0.809, g=0.12; CEW–C Δ=2.68 [−4.87,10.23], p=0.441, g=0.41.	CEW–CE Δ=0.87 [−5.45,7.19], p=0.772, g=0.12; CEW–C Δ=−2.62 [−16.27,11.04], p=0.682, g=−0.19

Conscientiousness	F(2,49)=3.43, p=0.040, η²=0.123	C n=9, CE n=8, CEW n=10	C n=8, CE n=13, CEW n=8	CEW–CE Δ=−0.38 [−7.90,7.15], p=0.917, g=−0.05; CEW–C Δ=−7.06 [−20.18,6.07], p=0.265, g=−0.53.	CEW–CE Δ=2.84 [−2.96,8.63], p=0.308, g=0.49; CEW–C Δ=7.63 [1.98,13.27], p=0.0129, g=1.42
Openness	F(2,50)=1.40, p=0.257, η²=0.053	C n=17, CE n=16, CEW n=7	C n=0, CE n=5, CEW n=11	CEW–CE Δ=2.49 [−4.96,9.94], p=0.464, g=0.40; CEW–C Δ=1.02 [−7.91,9.94], p=0.813, g=0.09.	CEW–CE Δ=−2.13 [−11.43,7.17], p=0.619, g=−0.24; CEW–C not estimable
Extraversion	F(2,49)=0.55, p=0.579, η²=0.022	C n=10, CE n=7, CEW n=7	C n=7, CE n=14, CEW n=11	CEW–CE Δ=0.29 [−7.15,7.72], p=0.934, g=0.04; CEW–C Δ=3.20 [−6.95,13.35], p=0.511, g=0.29.	CEW–CE Δ=0.62 [−6.04,7.27], p=0.847, g=0.08; CEW–C Δ=−4.60 [−16.83,7.64], p=0.422, g=−0.42
